# Seinäjoki Adult Asthma Study (SAAS): a protocol for a 12-year real-life follow-up study of new-onset asthma diagnosed at adult age and treated in primary and specialised care

**DOI:** 10.1038/npjpcrm.2015.42

**Published:** 2015-06-25

**Authors:** Hannu Kankaanranta, Pinja Ilmarinen, Terhi Kankaanranta, Leena E Tuomisto

**Affiliations:** 1 Department of Respiratory Medicine, Seinäjoki Central Hospital, Seinäjoki, Finland; 2 Department of Respiratory Medicine, University of Tampere, Tampere, Finland; 3 Police University College, Tampere, Finland

## Background

Asthma is characterised by variable symptoms of wheeze, shortness of breath, chest tightness and/or cough, and by variable expiratory flow limitation.^1^ Asthma can affect patients at any age with varying severities. Until recently, asthma has been considered to be a single, allergic, eosinophilic, T_H_2-mediated and glucocorticoid-responsive disease.^2,3^ Recently, cluster analyses have suggested that patients with asthma can be divided into different phenotypes, and the age at disease onset was found as a key differentiating factor between phenotypes.^2,3^ Later- or adult-onset disease is less associated with allergy than asthma beginning in childhood. Adult-onset phenotypes such as late-onset eosinophilic (often severe), exercise-induced, obesity-related and neutrophilic asthma have been proposed.^2,3^ Most publications on asthma have focused on allergic asthma starting in childhood.^2,3^ The symptoms in childhood are often transient, and approximately three out of four asthmatic children will outgrow their asthma.^4^ In contrast, the long-term prognosis of adult-onset asthma is not known, and only two^5,6^ follow-up studies of duration of 2–5.8 years have been published and suggest a less favourable outcome. Thus, long-term, real-life, follow-up studies with asthma patients treated in primary care are needed. Allergic childhood asthma can usually be treated well by inhaled glucocorticoids,^2–4^ whereas the need for different add-on therapies^7^ is common in adult-onset disease and the therapeutic response may remain insufficient.^2,3,7^ In adult patients with asthma, co-morbidities are common.^8,9^ Still, a single-disease paradigm dominates randomised controlled trials, health policy, delivery and guidelines.^8^ The current guidelines on diagnostics and treatment of asthma^1,10^ or asthma–chronic obstructive pulmonary disease (COPD) overlap syndrome^1,11,12^ offer us advice and information mainly on the epidemiology, risk factors, diagnostic criteria, (initial) pharmacotherapy and treatment of exacerbations. However, when it comes to exact recommendations on whether the diagnostic studies and the diagnosis of asthma should be made in primary practice, the recommendations, if they exist at all, are based on opinion rather than on evidence. Furthermore, a similar lack of information remains when specific questions on the organisation of the follow-up of chronically ill patients with asthma are asked. Examples of such questions are as follows: who should perform the follow-up checks? How often should these follow-up checks be performed? Exactly what follow-up tools should be used?

## Aims

The aim of this study is to increase the understanding on the diagnostics and diagnostic process, organisation of the long-term asthma care, therapeutic outcomes, prognosis and the factors affecting the prognosis of new-onset asthma diagnosed at adult age.

## Methods

### Study design, inclusion and exclusion criteria

**Figure 1:**
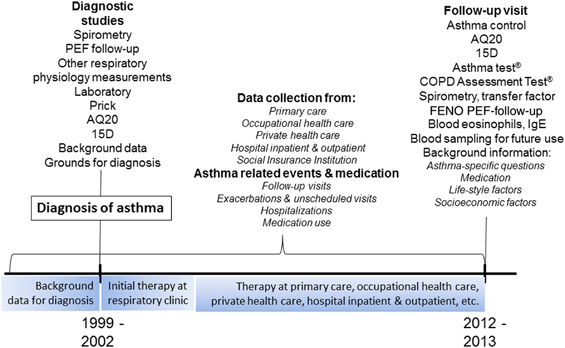
Schematic presentation of the Seinäjoki Adult Asthma Study. The original cohort (phase I) was collected between 6 October 1999 and 17 April 2002, and follow-up visit (phase II) was performed between 10 December 2012 and 31 October 2013.

Seinäjoki Adult Asthma Study (SAAS) is a single-centre (Department of Respiratory Medicine, Seinäjoki Central Hospital, Seinäjoki, Finland) 12-year follow-up study of a total cohort of 259 patients having new-onset asthma that was diagnosed at adult age. However, two patients were excluded because they were later found to have a previous diagnosis of asthma during childhood, leaving 257 patients in the original cohort. The study was divided in two parts (Figure 1): the collection of the original cohort (phase I) and follow-up visit (phase II). The original cohort was collected between 6 October 1999 and 17 April 2002. Patients were referred to the hospital by primary-care practitioners because of suspicion of asthma. Inclusion and exclusion criteria are shown in Table 1. Patients with simultaneous asthma and COPD were not excluded, and the study population includes patients who could be defined as having asthma–COPD overlap syndrome, even though the inclusion criteria for those patients are not exactly the same as currently used criteria for asthma–COPD overlap syndrome.^1,11,12^

After 12 years, patients were invited to a follow-up visit (phase II; 10 December 2012 and 31 October 2013) in which asthma status, co-morbidities (chronic rhinitis or obstructed nose, allergic rhinitis or conjunctivitis, diabetes, hypertension, coronary heart disease, COPD and any other patient-reported disease), medication (including medication to other diseases and the disease treated), control, severity and lung function were evaluated (Figure 1). In addition to the data gathered at these visits, data on asthma follow-up visits, exacerbations, hospitalisations, possible occupationally induced asthma and prescribed asthma medication were collected from hospital clinics, primary health care, occupational health care and private practices for the whole 12-year follow-up period. In addition, the use of medication that was realised, i.e., medication bought from pharmacy, will be retrieved. In addition to asthma-specific factors, data include occupational, lifestyle and socioeconomic factors at the follow-up visit.

### Ethical considerations and permissions

Phase I was originally designed as a registry serving as an asthma-related data exchange platform between primary and specialised care, as well as an asthma-related research registry. Phase I was part of hospital development projects (institutional permission TU 1114). No interventions outside normal clinical practice were carried out. All participants in the original cohort gave written informed consent to be included in the registry. With the development of a common regional electronic patient record system, the data exchange platform became unnecessary, but the research registry remained. The participants of the follow-up visit (phase II) gave written informed consent to the study protocol approved by the Ethics committee of Tampere University Hospital, Tampere, Finland (R12122).

### Setting and background data

The organisation of asthma care in general and especially in the Seinäjoki Central Hospital district has recently been described in detail.^13^ Asthma is a common disease needing community solutions.^14^ The actions of the Finnish Asthma Programme^14,15^ have been put into practice in the hospital district.^13,16^ The original cohort (phase I) represents novel adult asthma cases well; for example, the total number of new diagnoses of asthma at the study centre in 2001 was 133.^16^ Of those, 126 patients were recruited to phase I of this study, representing 94.7% of new diagnoses of asthma.

### The main planned outcomes

#### Prognosis of new-onset asthma diagnosed at adult age during a 12-year follow-up

The questions the clinician is facing in front of an adult patient with newly diagnosed asthma are as follows: what is the prognosis of adult-onset asthma in general and especially in this particular patient? Are there any prognostic markers that would help me in predicting the future and guide me in planning his/her future therapy? The primary outcome is the prognosis of asthma (remission, control and severity). However, evaluation of control at a single time point does not represent the true morbidity of asthma.^17^ Our intention is to characterise the true 12-year prognosis of asthma, i.e., cumulative burden of asthma-related events, which is the second primary outcome. Secondary outcomes include the following: exacerbations, hospitalisations and mortality because of asthma, multimorbidity, lung function and inflammatory cells (e.g., blood eosinophils and neutrophils), as well as inflammatory and other markers of interest^18–21^ in the pathogenesis of asthma such as interleukins, adipokines and periostin (Figure 1).

#### Diagnostics and follow-up of patients with adult-onset asthma in primary and specialised care

The diagnosis of asthma with all patients in the present study was confirmed by a respiratory specialist, but diagnostic studies for most patients were partly performed already in primary care. This gives us the opportunity to assess the proportion of diagnoses that could have been done already in primary care and the diagnostic tools that were able to provide sufficient information on the diagnosis of adult-onset asthma in primary care. All asthma-related follow-up visits over a 12-year period both in primary and specialised care are collected and evaluated. Thus, the impact of asthma follow-up visits on the outcome of asthma during a 12-year period can be assessed. This gives us a possibility to evaluate whether, e.g., the place or the performer, the timing or frequency of control visits and actions performed at the control visit can contribute to the outcome of asthma and how the care of chronic asthma should be organised.

### Statistical analysis

Statistical analysis involves the basic statistical tools for continuous, categorical or dichotomous variables such as *t*-test, nonparametric tests (e.g., Mann–Whitney) and analysis of variance. To evaluate the associations between variables, *χ*^2^-test, correlation matrixes and regression analysis will be used.^22,23^

Usually, the risk of asthma attacks is expressed as the total number of events per patient or as yearly incidence of events.^17^ However, this analysis does not take into account the timing of asthma attacks in relation to changes in the other factors in asthma care (e.g., change in medication). Even though Cox regression analysis can also involve time-dependent variables, it will be inevitable to develop new ways to express the risk of asthma attacks in relation to other changes in the condition of the patient. Therefore, more sophisticated statistical methods will be included to illustrate these connections. Cluster analyses of patients with asthma have suggested that patients with asthma can be divided into different phenotypes.^24–26^ However, the studies published thus far have been cross-sectional and analysed their subjects at one single time point or after only a short follow-up. Analysis of patient data with a long-term follow-up time with time-dependent incidents will require a more sophisticated way of performing cluster-type analysis. Thus, we will evaluate whether the long-term follow-up will identify new and/or different clusters among patients with adult-onset disease.

## Discussion

The characterisation of phenotypes of asthma is still in process.^2,3^ As the phenotypes have been characterised relatively recently (i.e., between 2008 and 2014),^24–26^ there has not been enough time for long-term follow-up studies to be conducted. Our recent published systematic literature review^27^ identified only one follow-up study^5^ of newly diagnosed adult-onset asthma lasting ⩾5 years. Another 2-year follow-up study of adult-onset asthma has been recently published.^6^ Thus, the present study will increase our knowledge on the long-term prognosis of new-onset asthma diagnosed at adult age. The exclusion criteria in most studies with asthma are current smoking or smoking history ⩾10 pack-years, as well as the presence of co-morbidities. However, the therapeutic response in patients with asthma who smoke remains insufficient.^28,29^ A recent survey^9^ indicated that over 60% of asthma sufferers have one or more additional co-morbidities. Furthermore, these co-morbidities associate with unscheduled asthma care among adults.^8,9^ The patient population generally included in clinical trials in asthma^7^ has been shown to represent only 1.3–5.4% of those obstructive seen by a generalist.^30^ In the present study, patients were not excluded because of smoking or any significant co-morbidity, suggesting that the study population more closely represents that seen by a generalist.

Asthma is a common disease needing community solutions.^14^ Early diagnosis, active treatment and self-management are not possible without the active role of primary-care professionals. The key for the implementation of the Finnish Asthma Programme^14,15^ was the primary-care network of local asthma co-ordinators (physicians and nurses) in local health-care centres.^13^ The Finnish Asthma Programme reduced, e.g., the number of asthma hospital days and morbidity.^15,31^ The published reports^15,31^ give indirect evidence to support the primary-care-centred organisation of care of chronic asthma, but more evidence is needed. The Finnish Asthma Programme was extensively used in the Seinäjoki Hospital district and has been evaluated.^13,16,32–34^ This allows us to evaluate the diagnostic process, as well as the follow-up visits made both in primary and specialised health care. According to the principles of the Finnish Asthma Programme,^13–15^ a hypothesis to be tested is that the diagnostic evaluations in patients with adult-onset asthma can be performed in primary care. Similarly, another hypothesis, supported also by the literature,^35^ is that specialised nurse-centred follow-up visits are important in the follow-up of most patients with adult-onset asthma.

## Figures and Tables

**Table 1:** Inclusion and exclusion criteria used in SAAS

Inclusion criteria	A diagnosis of new-onset asthma made by a respiratory specialist Diagnosis confirmed by at least one of the following objective lung function measurements:^a^ FEV_1_ reversibility in spirometry of at least 15% and 200 ml Diurnal variability (⩾20%) or repeated reversibility (⩾15%/60 l/min) in PEF follow-up A significant decrease in FEV_1_ (15%) or PEF (20%) in response to exercise or allergen A significant reversibility in FEV_1_ (at least 15% and 200 ml) or significant mean PEF change in response to a trial with oral or inhaled glucocorticoids Symptoms of asthma Age ⩾15 years
Exclusion criteria	Physical or mental inability to provide signed informed consent Diagnosis of asthma below the age of 15 years Of note: Patients with comorbidities, either other lung disease or any other significant disease, were not excluded Patients were not excluded because of smoking, alcohol use or any other lifestyle factor Respiratory symptoms or any other disease during childhood was not a reason to exclude patients, but a diagnosis of asthma at age <15 years was an exclusion criteria

Abbreviations: FEV_1_, forced expiratory volume in one second; PEF, peak expiratory flow; SAAS, Seinäjoki Adult Asthma Study.

a The objective lung function criteria reflect those of National and International Guidelines valid in 1999–2002 and may not exactly follow those valid at the moment.^1,10^

## Notes

HK reports personal fees and non-financial support from Almirall, personal fees from AstraZeneca, personal fees from Chiesi Pharma AB, personal fees from GlaxoSmithKline, personal fees and non-financial support from Boehringer-Ingelmheim, personal fees from Leiras-Takeda, personal fees from MSD (Merck Sharp & Dohme Corp.), personal fees from Novartis, personal fees from Mundipharma, personal fees from Medith, personal fees from Resmed Finland and non-financial support from Intermune, outside the submitted work. LET reports personal fees and non-financial support from Leiras-Takeda, personal fees and non-financial support from Mundipharma, personal fees and non-financial support from Novartis, and non-financial support from Boehringer-Ingelheim, outside the submitted work. The other authors declare no conflicts of interest.

## References

[bib1] Global Initiative for Asthma Global Strategy for Asthma Management and Prevention . Revised 2014. Available at www.ginasthma.org .

[bib2] Wenzel SE Asthma phenotypes: the evolution from clinical to molecular approaches Nat Med 2012 18 716 725 22561835 10.1038/nm.2678

[bib3] de Nijs SB Venekamp LN Bel EH Adult-onset asthma: is it really different? Eur Respir Rev 2013 22 44 52 23457164 10.1183/09059180.00007112 PMC9487439

[bib4] Bisgaard H Bønnelykke K Long-term studies of the natural history of asthma in childhood J Allergy Clin Immunol. 2010 126 187 197 20688204 10.1016/j.jaci.2010.07.011

[bib5] Rönmark E Lindberg A Watson L Lundbäck B Outcome and severity of adult onset asthma—report from the obstructive lung disease in northern Sweden studies (OLIN) Respir Med. 2007 101 2370 2377 17689949 10.1016/j.rmed.2007.06.011

[bib6] Westerhof GA Vollema EM Weersink EJ Reinartz SM de Nijs SB Bel EH Predictors for the development of progressive severity in new-onset adult asthma J Allergy Clin Immunol 2014 134 1051 1056 24954263 10.1016/j.jaci.2014.05.005

[bib7] Kankaanranta H Lahdensuo A Moilanen E Barnes PJ ‘Add-on therapy’ options in adults with asthma not adequately controlled by inhaled corticosteroids: a comprehensive review Respir Res 2004 5 17 15509300 10.1186/1465-9921-5-17 PMC528858

[bib8] Mercer SW Comorbidity in asthma is important and requires a generalist approach Prim Care Respir J 2014 23 4 5 24553822 10.4104/pcrj.2014.00012 PMC6442280

[bib9] Steppuhn H Langen U Keil T Scheidt-Nave C Chronic disease co-morbidity of asthma and unscheduled asthma care among adults: results of the national telephone health interview survey German Health Update (GEDA) 2009 and 2010 Prim Care Respir J 2014 23 22 29 24346826 10.4104/pcrj.2013.00107 PMC6442275

[bib10] Haahtela T Lehtimäki L Ahonen E Harju T Jartti T Kankaanranta H Update on current guidelines: asthma Duodecim 2013 129 994 995 23786112

[bib11] Global Initiative for Chronic Obstructive Lung Disease Global Strategy for the Diagnosis, Management and Prevention of COPD 2014 . Available at www.goldcopd.org .

[bib12] Kankaanranta H Harju T Kilpeläinen M Mazur W Lehto JT Katajisto M Diagnosis and pharmacotherapy of stable chronic obstructive pulmonary disease: the Finnish guidelines Basic Clin Pharmacol Toxicol 2015 116 291 307 25515181 10.1111/bcpt.12366 PMC4409821

[bib13] Tuomisto L Asthma programme in Finland—management of adult asthma as reflected by referral letters (Acta Universitatis Tamperensis) Tampere University Press: Tampere, FinlandTampere, Finland, 2010 . Available at http://tampub.uta.fi/handle/10024/59337/browse?value=Tuomisto%2C+Leena&type=author .

[bib14] Haahtela T Klaukka T Koskela K Erhola M Laitinen LA Asthma Programme in Finland: a community problem needs community solutions Thorax 2001 56 806 814 11562522 10.1136/thorax.56.10.806 PMC1745939

[bib15] Haahtela T Tuomisto LE Pietinalho A Klaukka T Erhola M Kaila M A 10 year asthma programme in Finland: major change for the better Thorax 2006 61 663 670 16877690 10.1136/thx.2005.055699 PMC2104683

[bib16] Tuomisto LE Erhola M Luukkaala T Puolijoki H Nieminen MM Kaila M Asthma Programme in Finland: did the use of secondary care resources become more rational Respir Med 2010 104 957 965 20207125 10.1016/j.rmed.2010.01.018

[bib17] Blakey JD Woolnough K Fellows J Walker S Thomas M Pavord ID Assessing the risk of attack in the management of asthma: a review and proposal for revision of the current control-centered paradigm Prim Care Respir J 2013 22 344 352 23817678 10.4104/pcrj.2013.00063 PMC6442819

[bib18] Ilmarinen P Kankaanranta H Eosinophil apoptosis as a therapeutic target in allergic asthma Basic Clin Pharmacol Toxicol 2014 114 109 117 24148899 10.1111/bcpt.12163

[bib19] Leivo-Korpela S Lehtimäki L Vuolteenaho K Nieminen R Kööbi L Järvenpää R Adiponectin is associated with dynamic hyperinflation and a favourable response to inhaled glucocorticoids in patients with COPD Respir Med 2014 108 122 128 24135487 10.1016/j.rmed.2013.08.016

[bib20] Ilmarinen P Moilanen E Erjefält J Kankaanranta H The polyamine spermine promotes survival and activation of human eosinophils J Allergy Clin Immunol 2015 10.1016/j.jaci.2014.12.1922 25649081

[bib21] Izuhara K Arima K Ohta S Suzuki S Inamitsu M Yamamoto K Periostin in allergic inflammation Allergol Int 2014 63 143 151 24662806 10.2332/allergolint.13-RAI-0663

[bib22] Kankaanranta T Nummi T Vainiomäki J Halila H Hyppölä H Isokoski M The role of job satisfaction, job dissatisfaction and demographic factors on physician’s interventions to switch work sector from public to private Health Policy 2007 83 50 65 17188394 10.1016/j.healthpol.2006.11.010

[bib23] Kankaanranta T Rissanen P The labour supply of registered nurses in Finland: the effect of wages and working conditions Eur J Health Econ 2009 10 167 178 18615259 10.1007/s10198-008-0116-3

[bib24] Haldar P Pavord ID Shaw DE Berry MA Thomas M Brightling CE Cluster analysis and clinical asthma phenotypes Am J Respir Crit Care Med 2008 178 218 224 18480428 10.1164/rccm.200711-1754OC PMC3992366

[bib25] Moore WC Meyers DA Wenzel SE Teague WG Li H Li X National Heart, Lung, and Blood Institute's Severe Asthma Research Program. Identification of asthma phenotypes using cluster analysis in the Severe Asthma Research Program Am J Respir Crit Care Med 2010 181 315 323 19892860 10.1164/rccm.200906-0896OC PMC2822971

[bib26] Siroux V Basagaña X Boudier A Pin I Garcia-Aymerich J Vesin A Identifying adult asthma phenotypes using a clustering approach Eur Respir J 2011 38 310 317 21233270 10.1183/09031936.00120810

[bib27] Tuomisto LE Ilmarinen P Kankaanranta H Prognosis of new-onset asthma diagnosed at adult age Respir Med 2015 10.1016/j.rmed.2015.05.001 26052036

[bib28] Polosa R Thomson NC Smoking and asthma: dangerous liaisons Eur Respir J 2013 41 716 726 22903959 10.1183/09031936.00073312

[bib29] Spears M McSharry C Chaudhuri R Weir CJ de Wet C Thomson NC Smoking in asthma is associated with elevated levels of corticosteroid resistant sputum cytokines—an exploratory study PLoS One 2013 8 e71460 23951170 10.1371/journal.pone.0071460 PMC3739804

[bib30] Herland K Akselsen J-P Skjønsberg OH Bjermer L How representative are clinical study patients with asthma or COPD for a larger ‘real life’ population of patients with obstructive lung disease Respir Med 2005 99 11 19 15672843 10.1016/j.rmed.2004.03.026

[bib31] Kauppi P Linna M Martikainen J Mäkelä MJ Haahtela T Follow-up of the Finnish Asthma Programme 2000-2010: reduction of hospital burden needs risk group rethinking Thorax 2013 68 292 293 22504963 10.1136/thoraxjnl-2011-201028

[bib32] Tuomisto LE Erhola M Kaila M Brander P Kauppinen R Puolijoki H The Finnish national asthma programme: communication in asthma care—quality assessment of asthma referral letters J Eval Clin Pract 2007 13 50 54 17286723 10.1111/j.1365-2753.2006.00645.x

[bib33] Tuomisto LE Kaila M Erhola M Asthma programme in Finland: comparison of adult asthma referral letters in 1994 and 2001 Respir Med 2007 101 595 600 16890421 10.1016/j.rmed.2006.06.010

[bib34] Tuomisto LE Järvinen V Laitinen J Erhola M Kaila M Brander P Asthma Programme in Finland: the quality of primary care spirometry is good Prim Care Respir J 2008 17 226 231 18830519 10.3132/pcrj.2008.00053 PMC6619905

[bib35] Martinez-Gonzalez NA Tandjung R Djalali S Huber-Geismann F Markun S Rosemann T Effects of physician-nurse substitution on clinical parameters: a systematic review and meta-analysis PLoS One 2014 9 e89181 24586577 10.1371/journal.pone.0089181 PMC3933531

